# Opposing Roles of CYP2E1 and ALDH2 in Binge Alcohol-Mediated Cerebellar Damage

**DOI:** 10.3390/ijms27156825

**Published:** 2026-07-30

**Authors:** Leon Ruiter-Lopez, Wiramon Rungratanawanich, Byoung-Joon Song

**Affiliations:** Section of Molecular Pharmacology and Toxicology, National Institute on Alcohol Abuse and Alcoholism, Bethesda, MD 20892, USA; leon.ruiterlopez@nih.gov (L.R.-L.); wiramon.rungratanawanich@nih.gov (W.R.)

**Keywords:** alcohol, cerebellum, oxidative stress, CYP2E1, ALDH2, post-translational modifications, neuronal damage, locomotor test

## Abstract

To explore the opposing roles between the toxic acetaldehyde-producing enzyme Cytochrome P450-2E1 (CYP2E1) and acetaldehyde-clearing protein mitochondrial Aldehyde Dehydrogenase-2 (ALDH2) in alcohol-mediated cerebellar damage, two separate experiments were conducted. Young Svj/129 wild-type (WT) vs. *Cyp2e1*-Knockout (KO) mice were administered consecutive doses of 5 g/kg/dose ethanol (EtOH) at 12 h intervals, and C57BL/6J WT vs. *Aldh2*-KO mice were administered 4 g/kg/dose EtOH at 12 h intervals. Control mice were given dextrose, and cerebella were collected one hour after the last ethanol dose. Cerebellar tissue extracts were used for immunoblot analyses, and frozen tissue sections were evaluated by confocal microscopy with Fluoro-Jade C (FJC) staining for damaged neurons. Rotarod locomotor function of another mouse group was evaluated 48 h after ethanol exposure. Representative immunoblots showed increased levels of oxidative post-translational modifications (PTMs) (e.g., p-Ser/Thr-Pro, Ac-Lys, and acrolein adducts) in EtOH-exposed WT mice, and FJC-staining images revealed more degenerated neurons with decreased locomotor activity in ethanol-exposed WT compared to the corresponding *Cyp2e1*-KO mice. However, elevated levels of oxidative PTMs were observed in alcohol-exposed *Aldh2*-KO mice with increased damaged neurons stained with FJC. Our results show that CYP2E1 is a contributing factor, while ALDH2 shows a protective function, demonstrating their opposing roles in binge alcohol-mediated cerebellar degeneration.

## 1. Introduction

Excessive alcohol consumption has detrimental effects on human health, impacting multiple organ systems and contributing to a variety of diseases. One such affected organ is the brain, where chronic alcohol intake negatively affects the regions involved in motivation, memory, decision-making, impulse handling, attention, motor coordination or balance control, and sleep regulation, as well as possibly impacting other cognitive and executive functions [1,2]. Binge alcohol consumption is also dangerous; if the amount of alcohol in the bloodstream is too great, basic life-support functions, including the heart–lung system, may shut down, and permanent brain damage or sudden death can occur [3]. In fact, binge alcohol consumption is attributable to more than two-thirds of alcohol-induced toxicity and associated tissue damage, while it is responsible for more than three-fourths of the total cost associated with alcohol-related socioeconomic burdens in 2010 [4,5].

One of the key brain structures adversely impacted by alcohol toxicity is the cerebellum. Acute and chronic alcohol intoxication symptoms such as loss of balance and coordination [6,7], involuntary eye movements, and hypotonia [8] are all related to impairments of the cerebellum, known as alcohol-mediated cerebellar dysfunction and degeneration. Yet it is often understudied when studying cerebral alcohol metabolism and alcohol-induced cerebellar damage, possibly due to a historical underestimation of cerebellar function compared to hippocampus-related learning and cognitive impairment, standardized imaging practices that exclude the cerebellum [9], and a major focus on reward and addiction pathways. However, the cerebellum (containing granule neurons and Purkinje cells) expresses the classical ethanol metabolizing enzymes catalase and cytochrome P450-2E1 (CYP2E1), which is inducible by alcohol exposure [10,11], and is sensitive to oxidative stress-mediated damage, as shown by its selective degeneration from chronic alcohol intake [12]. In fact, morphological and histological study of post-mortem autopsied human brains showed that approximately 40% of people with alcohol use disorder (AUD) suffer from cerebellar atrophy [13]. Although the molecular mechanisms of cerebellar atrophy are relatively unknown, they could result from poor alcohol-associated nutrition, including various essential vitamins and minerals. In addition, alcohol-mediated elevation of CYP2E1 could contribute to increased oxidative and nitrative stress, oxidative post-translational modifications (PTMs), mitochondrial dysfunction with decreased energy supply, and elevated necroptosis of hippocampal and cerebellar cells [12,14]. This problem is particularly exacerbated by the accumulation of toxic metabolites, including acetaldehyde and other lipid aldehydes, which makes the involvement of aldehyde dehydrogenase 2 (ALDH2) a crucial regulator of these toxic metabolites.

Within the cerebellum, ethanol (EtOH) is primarily metabolized through enzymatic pathways, with approximately 60% metabolized by catalase and 20% by CYP2E1 [15]. However, these proportions are not universal and may vary by species, ethanol concentration, and exposure paradigm; they were not measured in the present cerebellar samples. This metabolic pattern differs from hepatic ethanol metabolism, where alcohol dehydrogenase (ADH) is the predominant first-step enzyme that oxidizes ethanol to acetaldehyde, followed by ALDH2-mediated conversion of acetaldehyde to acetate. In contrast, ADH expression and functional activity are relatively limited in the brain, making catalase and inducible CYP2E1 more prominent contributors to local acetaldehyde generation during alcohol exposure, particularly under binge-drinking conditions. Moreover, reactive oxygen species (ROS) production through CYP2E1-mediated cerebral ethanol metabolism also becomes important in producing various forms of oxidative PTMs, leading to cell/brain damage, as shown in this study and other reports. However, the role of CYP2E1 can become more important due to its alcohol-mediated induction with potential suppression of catalase activity [10,14,16,17,18]. Under conditions of excessive alcohol exposure, as occurs during binge drinking, there is an increased reliance on the CYP2E1 enzyme to oxidatively metabolize EtOH with generation of ROS and toxic acetaldehyde. It has been seen that CYP2E1 protein and activity were significantly increased by ethanol in the cerebellum [17,19,20]. In addition, the enhanced levels of CYP2E1 were found in the post-mortem brains of people with AUD [21].

CYP2E1 metabolizes EtOH into acetaldehyde with the production of ROS, which can activate inflammation via stimulating NF-kB, a critical redox-sensitive transcription factor for pro-inflammatory cytokines and inducible nitric oxide synthase (iNOS) [22], which generates nitric oxide (NO). In addition, CYP2E1-mediated ROS can stimulate PTMs of cellular biomolecules such as DNA, lipids, and proteins. In the liver, CYP2E1 is partially responsible for the alcohol-mediated hepatotoxicity [23,24] through production of ROS, oxidative stress, and lipid peroxidation [25]. Enhanced ROS in the presence of NO produces more cytotoxic peroxynitrite, which contributes significantly to oxidative/nitrative stress, various PTMs, mitochondrial dysfunction, endoplasmic reticulum (ER) stress, and tissue damage [26,27] since these oxidatively modified proteins in the mitochondria and ER [28] usually become inactivated and do not carry out their intended functions.

In addition, alcohol metabolism can alter cellular acetylation status through disruption of nicotinamide adenine dinucleotide (NAD^+^) homeostasis. NAD^+^-dependent deacetylases, known as sirtuins, play critical roles in regulating protein deacetylation/acetylation. Several sirtuin isoforms (SIRTs), including SIRT1 and SIRT3 [29,30], have been shown to be suppressed during binge and chronic alcohol exposure in the liver. However, the role of sirtuins in alcohol-induced neurotoxicity, particularly within the cerebellum, remains poorly understood.

ALDH2′s role in attenuating the toxic effects of elevated acetaldehyde is essential in cellular defense against mitochondrial dysfunction and tissue injury. The acetaldehyde is converted to acetate to neutralize its toxicity as it is later broken down to H_2_O and CO_2_ [31]. In addition, ALDH2 is involved in the detoxification of highly reactive lipid aldehydes, such as malondialdehyde (MDA), acrolein (ACR), and 4-hydroxynonenal (4-HNE) produced from lipids under oxidative stress conditions and acts as a protective or defensive protein against multiple disease states associated with AUD {e.g., alcohol-associated liver disease (ALD) [14,28,32,33] and alcohol-related brain damage (ARBD) [7,12,34,35]}.

However, the involvement of ALDH2 can vary. After chronic exposure to alcohol, the strength of ALDH2 as a mediator of acetaldehyde is weakened, possibly due to oxidative modifications of its active site Cys and other residues [32]. Many East Asian people have a defective ALDH2*2 mutant allele, resulting in its inactivation and a flushing response after alcohol intake [36,37]. In mice, genetic deletion of *Aldh2* gene or *Aldh2*-knockout (KO) contributes to increased sensitivity to alcohol-related DNA damage and tissue injury compared with the wild-type (WT) counterparts [38,39,40,41,42].

Previous research indicates that CYP2E1-driven metabolism of alcohol propagates cerebellar damage [17] while the activation of ALDH2 by Alda-1 [43,44] serves as neuroprotective. Therefore, we hypothesize that reducing CYP2E1 activity while protecting ALDH2’s role in oxidative ethanol metabolism may confer further protection against alcohol-mediated cerebellar neurodegeneration.

## 2. Results

### 2.1. Decreased Cerebellar Damage Observed with Fluoro-Jade C Staining in Cyp2e1-KO Mice

**Figure 1:**
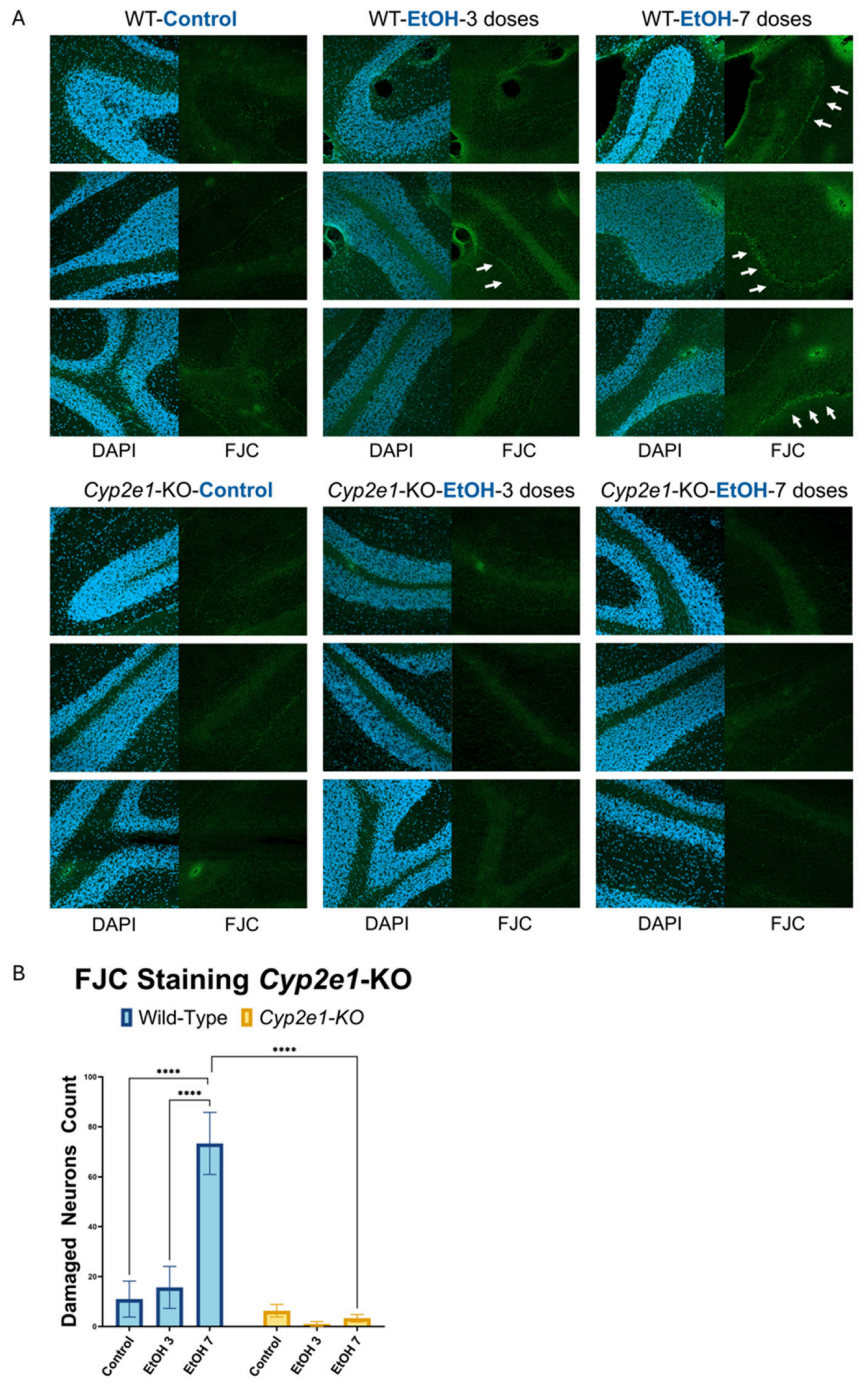
Decreased levels of FJC-stained damaged cells in the cerebella of *Cyp2e1*-KO mice after binge alcohol exposures. (**A**) Representative FJC-stained images of cerebellar sections from WT and *Cyp2e1*-KO mice treated with dextrose control, 3, or 7 doses of binge ethanol were analyzed by confocal microscopy (10× magnification). Cell nuclei were counter-stained with DAPI (4′,6′-diamidino-2-phenyl indole). Arrows indicate FJC-positive damaged neuronal cells. (**B**) Quantification of FJC-positive damaged neurons. Data are presented as the mean ± SD. Statistical analysis was performed by ordinary two-way ANOVA followed by Tukey’s multiple-comparisons test. Significance indicators shown in the graph reflect Tukey-adjusted pairwise comparisons. Complete ANOVA results, including genotype effects, ethanol exposure/dose effects, interaction terms, and post hoc comparisons, are provided in Supplementary Table S1. **** *p* < 0.0001.

Cerebellar damage following binge alcohol exposure was evaluated using FJC fluorescence staining to identify neurodegenerative cells. Compared to dextrose-treated control, mice subjected to three or seven binge alcohol doses displayed progressive increases in neuronal damage in the WT mice, with greater numbers of visibly damaged neurons corresponding to the increased number of binge alcohol exposures (Figure 1). In contrast, *Cyp2e1*-KO mice demonstrated markedly lower levels of neuronal damage across all tested doses. The most significant difference between *Cyp2e1*-KO and WT was observed after seven binge doses (Figure 1).

In this analysis, there were significant effects of genotype, F(1,12) = 83.61, *p* < 0.0001, ethanol exposure/dose, F(2,12) = 37.30, *p* < 0.0001, and genotype × ethanol interaction, F(2,12) = 38.92, *p* < 0.0001 (S1A).

### 2.2. Descriptive Immunoblot Analysis of Selected PTMs and VDAC Abundance in Alcohol-Exposed WT and Cyp2e1-KO Mice

**Figure 2:**
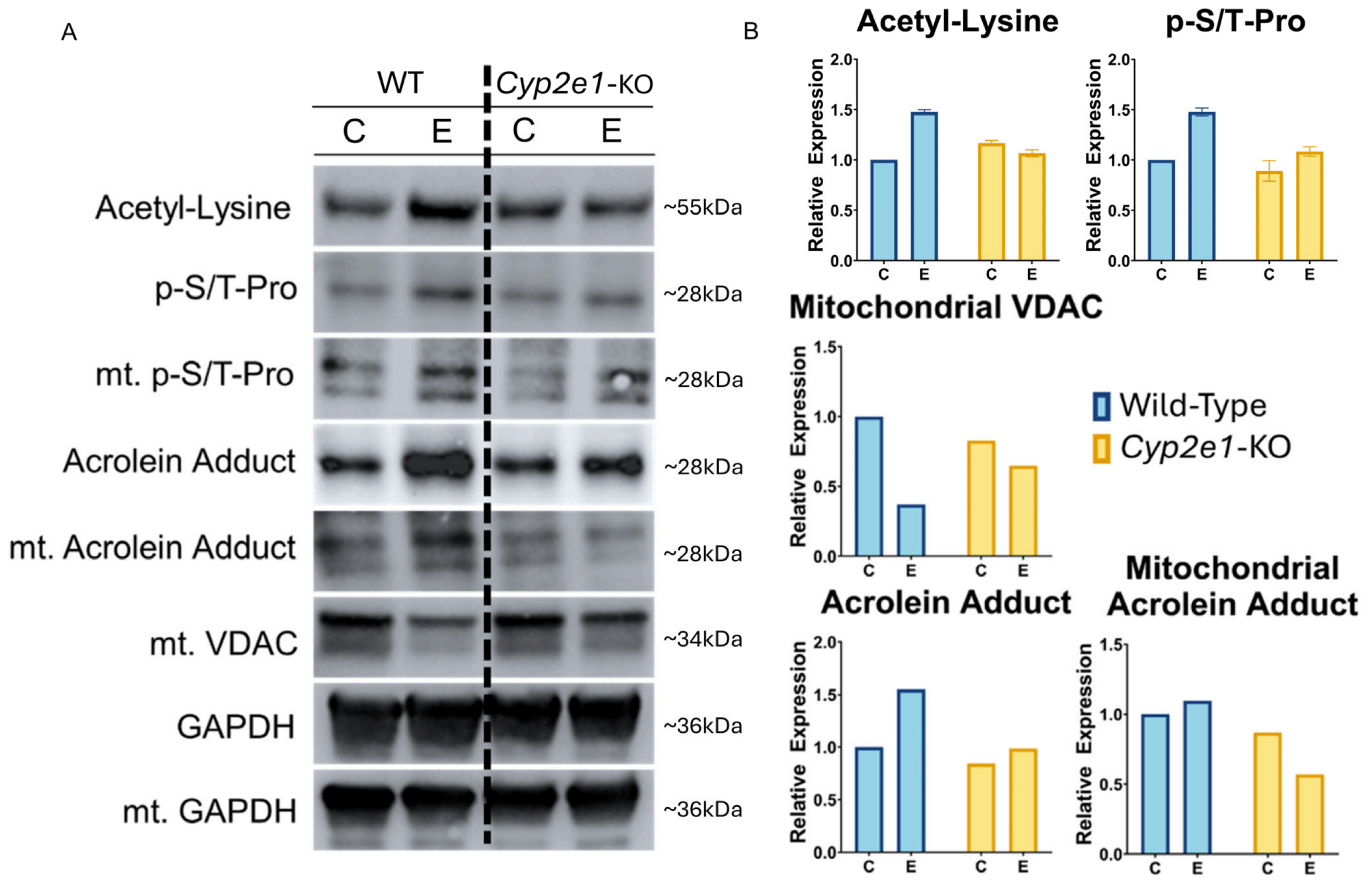
Representative immunoblot analysis of oxidative PTMs in alcohol-exposed WT and *Cyp2e1*-KO mice. (**A**) Cerebellar extracts were analyzed by immunoblotting for the indicated target proteins and PTMs in WT and *Cyp2e1*-KO mice after exposure to 3 doses of dextrose control (C) or ethanol (E). VDAC was used as a mitochondrial marker, and GAPDH was used as a loading control. (**B**) Densitometric visualization of the respective immunoblots. Because cerebellar lysates were pooled within each experimental group before immunoblotting, densitometry is presented descriptively.

Binge alcohol exposure increased PTMs, such as acetylation (Ac-Lys), mitogen-activated protein kinase-related phosphorylated proteins (p-S/T-Pro), and acrolein adducts, in total extracts and mitochondrial fractions (mt) in WT mice (Figure 2). Immunoblot analysis of the indicated target proteins and PTMs revealed qualitative changes, reflecting increased oxidative stress and decreased mitochondrial protein levels. Examination of mitochondrial health through the levels of Voltage-Dependent Anion Channel (VDAC) demonstrated marked decrements in alcohol-exposed WT, suggesting compromised mitochondrial integrity and neuronal health (Figure 2).

In contrast, *Cyp2e1*-KO mice displayed lower levels of PTMs, specifically phosphorylated serine/threonine residues (p-S/T-Pro) and acetylated proteins (Ac-Lys), with an improved mitochondrial health marker (mt-VDAC) compared to WT mice under ethanol conditions (Figure 2).

### 2.3. Decreased Rotarod Performance in Ethanol-Exposed WT Mice but Not in Cyp2e1-KO Counterparts

**Figure 3:**
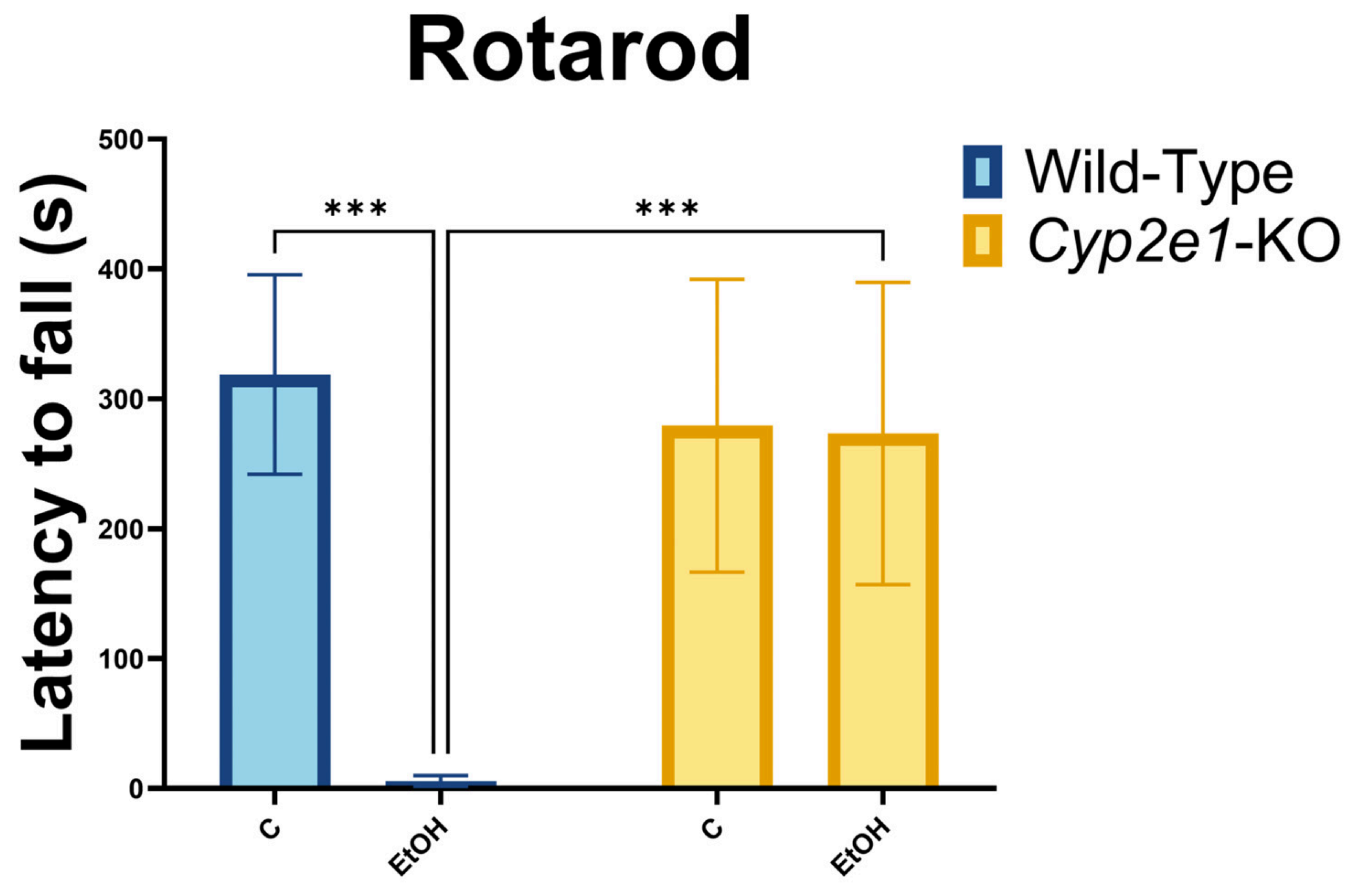
Greater impairment of rotarod performance in WT mice compared with *Cyp2e1*-KO mice after alcohol exposure. WT and *Cyp2e1*-KO mice were evaluated under control conditions or after exposure to 5 binge ethanol doses. Rotarod performance was measured as latency to fall. Data are presented as the mean ± SD. Statistical analysis was performed by ordinary two-way ANOVA followed by Tukey’s multiple-comparisons test. Significance indicators shown in the graph reflect Tukey-adjusted pairwise comparisons. Complete ANOVA results, including genotype effects, ethanol treatment effects, interaction terms, and post hoc comparisons, are provided in Supplementary Table S1. *** *p* < 0.001.

Rotarod performance tests were used to assess motor coordination, balance, and related functions shared by the cerebellum [45] and other brain regions following exposure to control and alcohol conditions. WT mice exhibited marked impairments, with difficulty maintaining control performance across several trials after the five doses of binge alcohol exposure (Figure 3). In contrast, the age- and sex-matched *Cyp2e1*-KO mice demonstrated robust performance, consistently reflecting proper balance and coordination on the rotarod, unaffected by alcohol exposure.

For the rotarod statistical analysis, there were significant effects of genotype, F(1,20) = 8.852, *p* = 0.0075, ethanol treatment, F(1,20) = 17.26, *p* = 0.0005, and genotype × ethanol interaction, F(1,20) = 16.00, *p* = 0.0007.

### 2.4. Increased Cerebellar Damage in Alcohol-Exposed Aldh2-KO Mice

**Figure 4:**
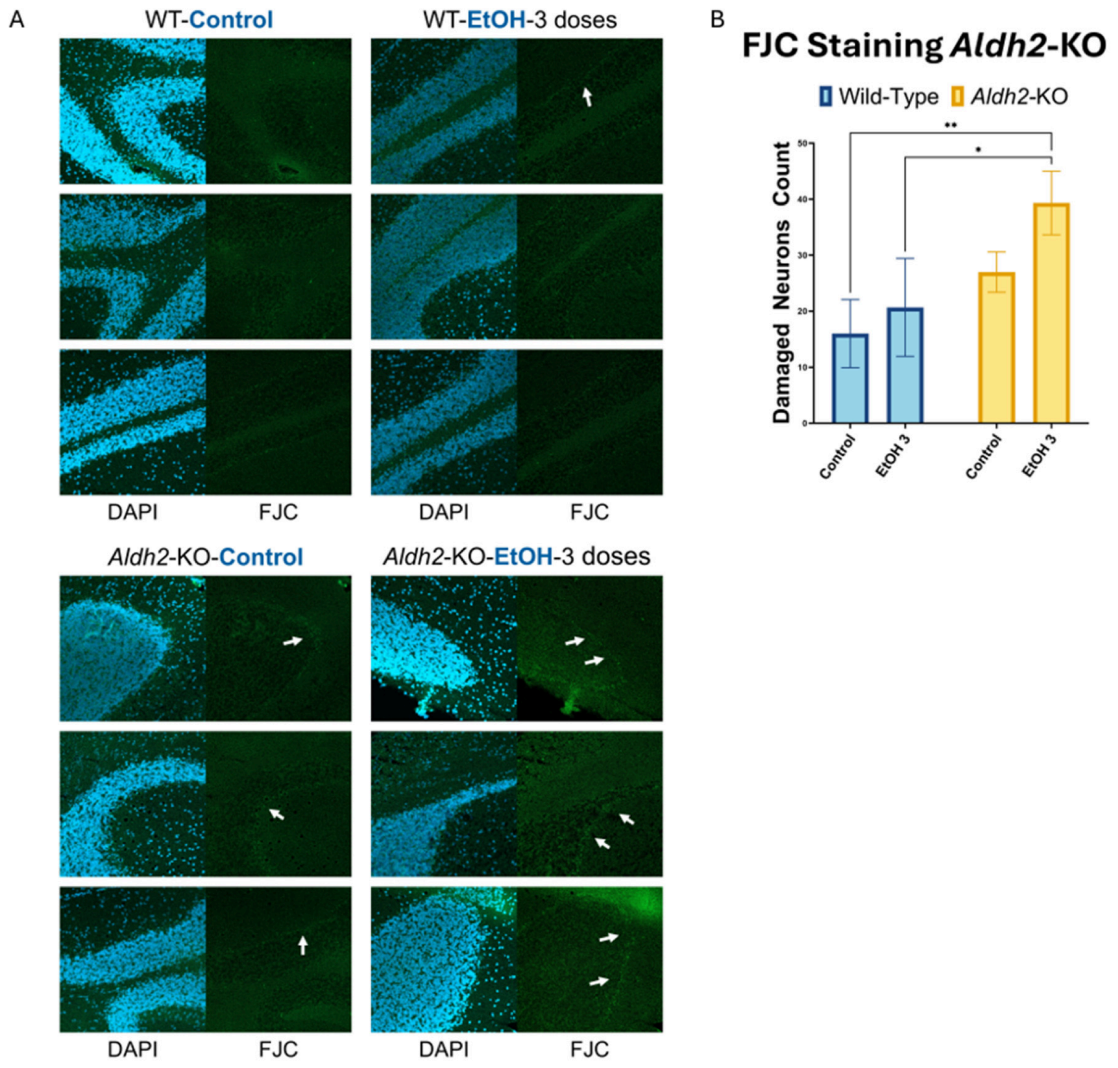
Increased cerebellar neurodegeneration in alcohol-exposed *Aldh2*-KO mice. (**A**) Representative FJC-stained images of cerebellar sections from WT and *Aldh2*-KO mice treated with dextrose control or 3 doses of binge ethanol were analyzed by confocal microscopy (10× magnification). Cell nuclei were counter-stained with DAPI. Arrows indicate FJC-positive damaged neuronal cells. (**B**) Quantification of FJC-positive damaged neurons. Data are presented as the mean ± SD. Statistical analysis was performed by ordinary two-way ANOVA followed by Tukey’s multiple-comparisons test. Significance indicators shown in the graph reflect Tukey-adjusted pairwise comparisons. Complete ANOVA results, including genotype effects, ethanol treatment effects, interaction terms, and post hoc comparisons, are provided in Supplementary Table S1. * *p* < 0.05, ** *p* < 0.01.

To study the protective role of ALDH2, cerebellar damage following binge alcohol exposure was evaluated by confocal microscopy evaluating FJC fluorescence staining for damaged cells. After three binge ethanol doses, *Aldh2*-KO mice showed significantly greater numbers of FJC-positive damaged neurons compared with ethanol-exposed WT mice (Figure 4). FJC staining also qualitatively increased after ethanol exposure in *Aldh2*-KO mice, although the within-genotype differences did not reach statistical significance.

In its statistical analysis, there were significant main effects of genotype, F(1,8) = 16.64, *p* = 0.0035, and ethanol treatment, F(1,8) = 5.464, *p* = 0.0476, but no significant genotype × ethanol interaction, F(1,8) = 1.111, *p* = 0.3226.

### 2.5. Descriptive Immunoblot Analysis of Selected Post-Translational Modifications in Alcohol-Exposed WT and Aldh2-KO Mice

**Figure 5:**
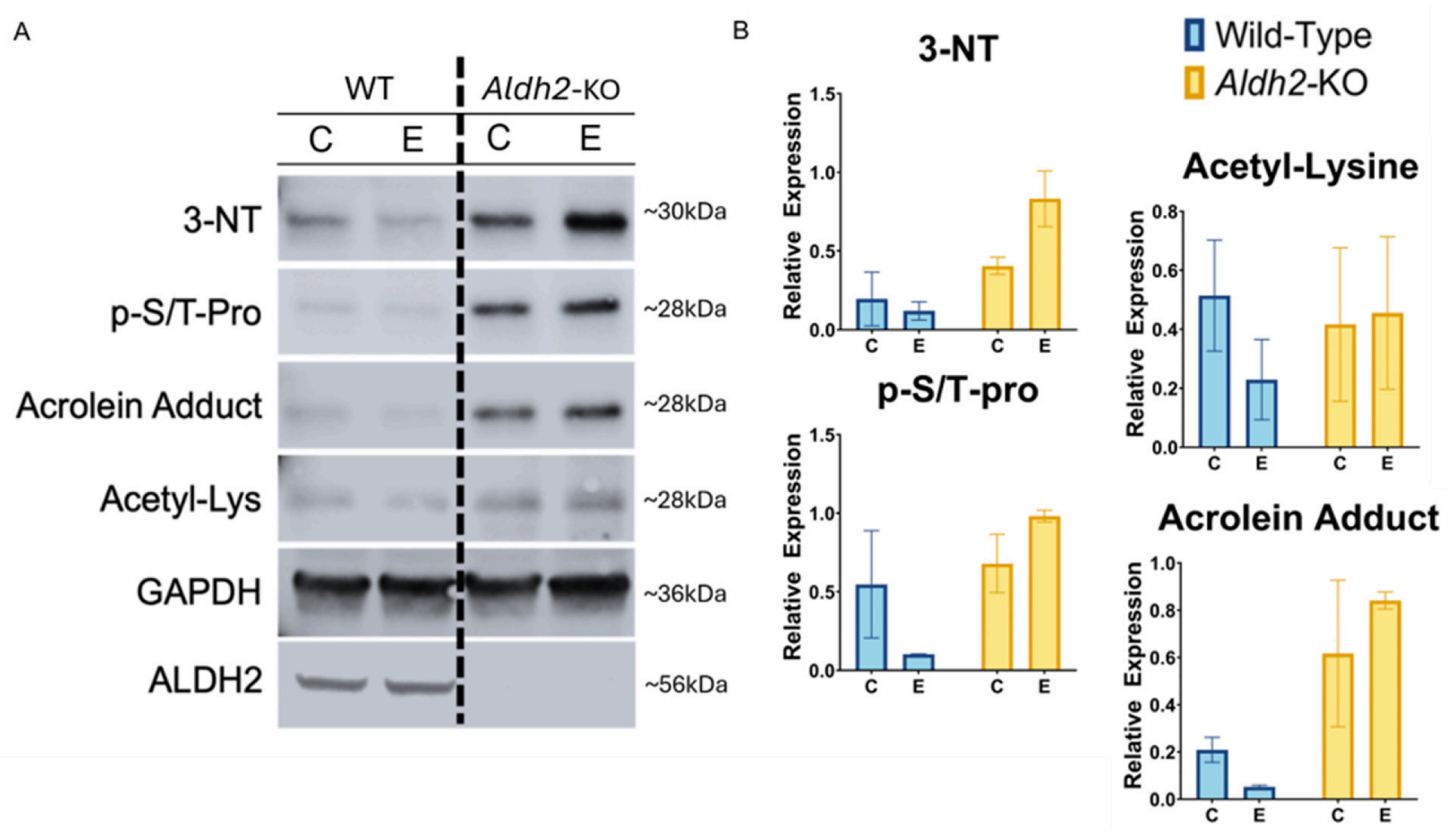
Representative immunoblot analysis of oxidative PTMs in alcohol-exposed WT and *Aldh2*-KO mice. (**A**) Cerebellar extracts were analyzed by immunoblotting for the indicated target proteins and PTMs in WT and *Aldh2*-KO mice after exposure to 3 doses of dextrose control (C) or ethanol (E). GAPDH was used as a loading control. (**B**) Densitometric visualization of the respective immunoblots. Because cerebellar lysates were pooled within each experimental group before immunoblotting, densitometry is presented descriptively.

For comparison with *Aldh2*-KO, age-matched young C57BL/6J black WT mice were used. Our previous reports showed that these C57BL/6J WT mice are more resistant to increases in oxidative PTMs, leading to gut and hippocampal damage after binge alcohol exposure [46]. In pooled *Aldh2*-KO samples, oxidative PTMs were overall heightened after alcohol exposure (Figure 5). Increases in 3-NT (nitration), acetyl-Lys (acetylation), and p-S/T-Pro phosphorylation, occurring via activated MAP kinase, which is detrimental and neurotoxic [47], were observed. The levels of acrolein adducts, which can also be damaging [48,49], were also increased in *Aldh2*-KO mice compared to the WT mice after alcohol exposure (Figure 5). In addition, motor coordination was evaluated via rotarod performance tests. However, our rotarod test results showed no significant differences in the locomotor function between WT and *Aldh2*-KO mice regardless of treatment with dextrose control or five doses of binge alcohol.

### 2.6. Minor Qualitative Changes in the Levels of Sirtuin Isoforms in the Cerebellum Following Ethanol Exposure

**Figure 6:**
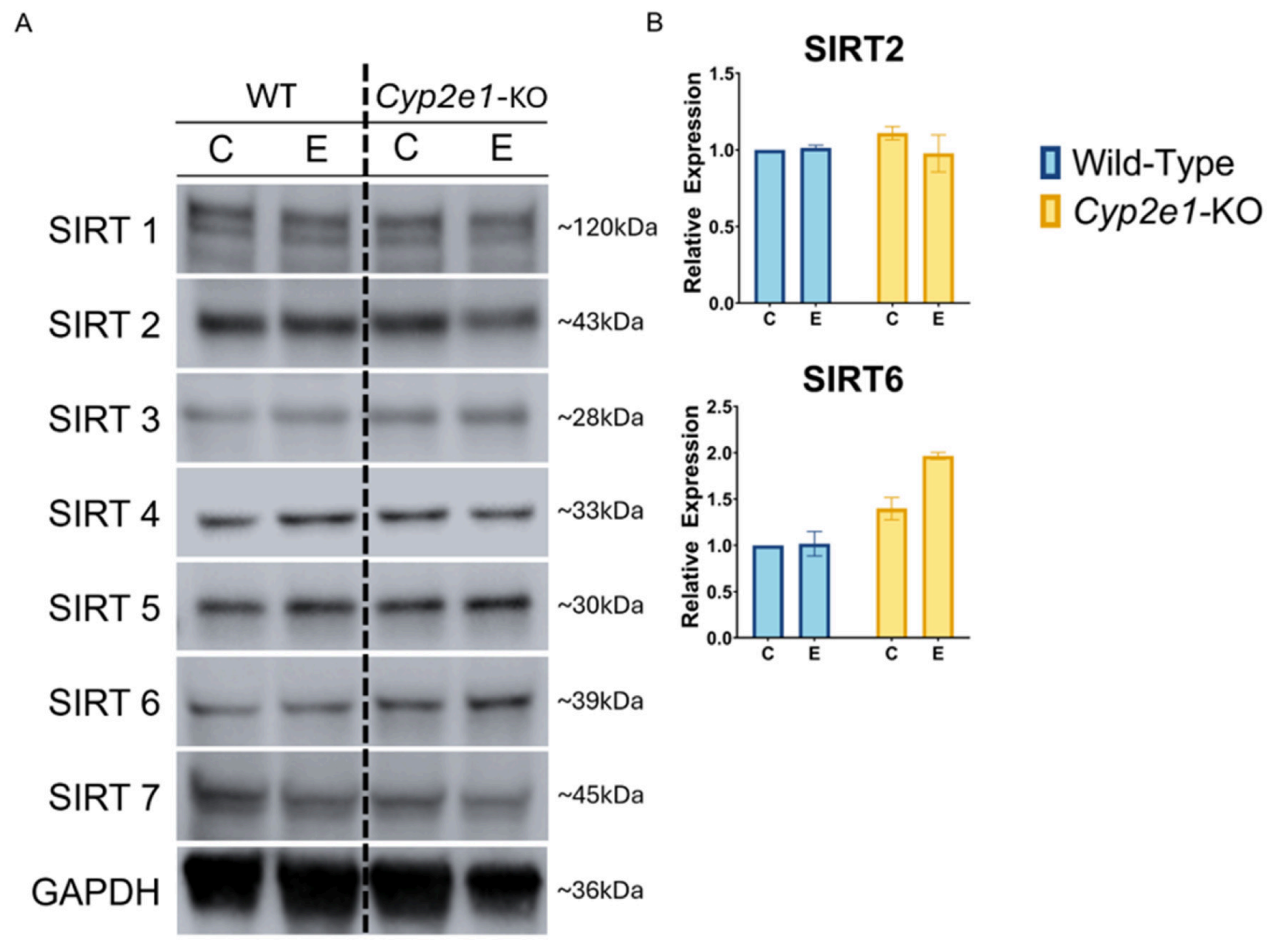
Representative immunoblot analysis of sirtuin isoforms in cerebellar tissues. (**A**) Sirtuin isoforms were analyzed by immunoblotting in cerebellar tissues from WT and *Cyp2e1*-KO mice after exposure to 3 doses of dextrose control (C) or ethanol (E). GAPDH was used as a loading control. (**B**) Densitometric visualization of the respective immunoblots. Because cerebellar lysates were pooled within each experimental group before immunoblotting, densitometry is presented descriptively.

Since we observed the elevated acetylated proteins (Figure 2 and Figure 5) in the current study, we further assessed the levels of sirtuin isoforms, NAD^+^-dependent deacetylases, in the cerebellum following exposure to dextrose or ethanol, since they were known to be involved in alcohol-associated liver disease in mice [30,50,51]. After confirming equal protein loading with the housekeeping protein GAPDH, we observed that the protein concentrations of SIRT isoforms 1, 2, 3, 5, and 7 remained unchanged across three doses of alcohol-treated groups and control conditions in both WT and *Cyp2e1*-KO mice (Figure 6). The levels of Sirt 6 did not change after alcohol exposure, although WT mice showed lower levels compared to those in *Cyp2e1*-KO mice.

## 3. Discussion

Alcohol-induced neurotoxicity has long been recognized as a major consequence of chronic and binge alcohol consumption, with the cerebellum emerging as a vulnerable brain region [1,2], although the underlying mechanisms of cerebellar degeneration have not been actively studied. In the central nervous system (CNS), acetaldehyde, a primary metabolite of oxidative ethanol metabolism via catalase and CYP2E1 produced locally in the CNS [15], has been shown to exert neurotoxic effects, particularly because acetaldehyde does not readily cross the blood–brain barrier and therefore must be generated in situ [52]. Research has highlighted acetaldehyde’s ability to react with structural and functional proteins, contributing to neuronal damage [53]. Numerous studies have also reported that oxidative stress, which can be induced by oxidative ethanol metabolism and immune cell activation, plays a significant role in alcohol-mediated neurotoxicity [54,55]. Clinically, cerebellar degeneration has been observed in a substantial proportion of individuals with chronic AUD, underscoring the selective vulnerability of this brain region to ethanol-induced toxicity [56,57]. The cerebellum’s susceptibility to alcohol may therefore be attributed, at least in part, to localized oxidative stress stemming at least in part from increased production of acetaldehyde [10] and CYP2E1-derived ROS [23,28], leading to detrimental PTMs and neuronal dysfunction that resemble mitochondrial dysfunction and signaling abnormalities observed in other neurodegenerative diseases [58].

Historically, mechanistic insight into alcohol-mediated tissue injury has largely originated from studies in the liver. CYP2E1 is another key enzyme in hepatic ethanol metabolism, particularly in the context of chronic and/or binge alcohol exposure and contributes significantly to ALD and metabolic dysfunction-associated fatty liver disease [59,60]. Its induction by ethanol leads to increased production of ROS and reactive metabolites, contributing to oxidative stress and subsequent PTMs, such as oxidation, nitration, phosphorylation, acetylation, etc., and impaired autophagy [12,28,61,62]. These PTMs usually disrupt cellular redox homeostasis and are involved in stimulating mitochondrial dysfunction, endoplasmic reticulum stress, and tissue injury, including neurodegeneration [14]. Despite this extensive hepatic literature, the extent to which similar CYP2E1-driven toxicity mechanisms operate within the cerebellum, and its underlying damaging mechanisms remain incompletely understood, representing a critical gap in understanding alcohol-induced cerebellar degeneration.

Beyond its effects in the liver, CYP2E1 activity has also been implicated in disrupting intestinal barrier integrity and function [63]. Ethanol-induced CYP2E1 expression in the gut promotes oxidative stress and PTMs, leading to degradation of tight junction and adherens junction proteins, enterocyte apoptosis, and increased intestinal permeability [64,65]. This “gut leakiness” facilitates endotoxemia and systemic inflammation, which further exacerbates hepatic injury through the gut–liver axis, as reported with *Aldh2*-KO mice [66]. Importantly, emerging evidence suggests that similar mechanisms may influence the CNS through a gut–brain axis. Based on our recent results [46], *Aldh2*-KO mice developed gut leakiness and subsequent brain (hippocampal) damage following exposure to three consecutive binge alcohol doses (4 g/kg/dose at 12 h intervals), at least in part through circulating inflammatory mediators, including endotoxin LPS, that can influence neuroinflammatory and neurodegenerative processes [67,68].

While ADH plays a predominant role in hepatic ethanol metabolism, its expression and functional activity in the brain are very limited [15]. Consequently, following alcohol exposure, ethanol-inducible CYP2E1 assumes a more prominent role in cerebral ethanol metabolism despite its inherent propensity to generate harmful ROS and acetaldehyde. In addition, all ALDH isozymes, including mitochondrial ALDH2, contain the active site Cys residue [32], which is easily oxidized and inactivated under oxidative stress, as shown previously in AUD people and alcohol-exposed rodents [26,27,69]. This metabolic shift and inactivation of protective ALDH enzymes may render the brain particularly vulnerable to oxidative injury since the brain possesses lower amounts of glutathione and antioxidant enzymes compared to those of the liver [70,71], and the oxidative conditions can be worsened after or during binge-drinking episodes.

Consistent with this framework, our study demonstrates that *Cyp2e1*-KO mice exhibit markedly reduced oxidative stress in the cerebellum and resistance to cerebellar damage following binge alcohol exposure. This protection is evidenced by a significant reduction in the quantity of FJC-stained damaged neurons between *Cyp2e1*-KO and WT observed after seven binge doses, illustrating the contributing role of CYP2E1 in promoting cerebellar neuronal damage during excessive alcohol exposure and metabolism (Figure 1). This was further exemplified by the preserved levels of VDAC, highlighting the role of CYP2E1 in the mitochondria through ROS production and PTMs (Figure 2), and decreased PTMs, including MAPK-mediated phosphorylation, acetylation, and acrolein adduct proteins, in alcohol-exposed *Cyp2e1*-KO mice compared with WT counterparts. Decreasing levels of acetylated proteins are essential as the usual hyper-acetylation effects seen in post-alcohol exposure can lead to enzyme inactivation, impaired autophagy, suppressed protein quality, altered transcriptional regulation, and disrupted cytoskeletal structure, as well as other detrimental effects [72,73,74]. Similarly, decreased p-S/T-Pro phosphorylation is also beneficial since its MAP kinase activity can create unintended cell death-related signaling and neurotoxicity [47]. Additionally, acrolein adducts, which can also damage neuronal cells [48,49], were reduced in *Cyp2e1*-KO mice even after alcohol exposures. These results reveal decreased oxidative PTMs in the absence of CYP2E1, which typically arise from increased ROS production during CYP2E1-mediated alcohol metabolism [75]. Together with the locomotor function assessments, these results indicate that CYP2E1 deficiency effectively prevents the deterioration of motor coordination and balance typically observed in WT mice following binge alcohol exposures (Figure 3), further corroborating the contributing role of CYP2E1 in alcohol-mediated cerebellum-associated dysfunction. It must be noted, though, that the molecular findings should be interpreted as early injury-associated changes rather than direct temporal correlates of the later rotarod outcomes, as molecular and behavioral outcomes were assessed at different post-ethanol time points.

Importantly, this pathogenic role of CYP2E1 contrasts with endogenous aldehyde-detoxifying systems, such as mitochondrial ALDH2, which function to prevent aldehyde accumulation and oxidative injury during ethanol metabolism. Our results with *Aldh2*-KO mice showed increases in PTMs, neurotoxic MAPK-mediated protein phosphorylation [47], nitration, and acrolein adduct formation upon alcohol exposure (Figure 5). In comparison to *Aldh2*-KO mice, however, it is important to note that C57BL/6J WT mice are relatively resistant to increases in PTMs even after binge alcohol exposure in both the current study and previous report [46], suggesting a protective or defensive role of ALDH2; however, because the WT and *Aldh2*-KO mice were originally obtained from different sources and were not littermates, source- or colony-related effects cannot be completely excluded. In relation to the motor function test results, we did not observe significant differences in rotarod performance among control- and alcohol-exposed WT and *Aldh2*-KO mice. The lack of detectable behavioral differences may reflect several experimental factors, including the lower ethanol dose used in the Aldh2 experiment (4 g/kg/dose vs. 5 g/kg/dose in the Cyp2e1 experiment), the different genetic backgrounds (C57BL/6J vs. Svj/129), and the later time point of rotarod assessment after the final ethanol dose (72 h vs. 48 h, respectively). Therefore, the absence of a significant rotarod effect in the Aldh2 groups should be interpreted within the specific conditions of this experiment. Because the CYP2E1 and ALDH2 experiments were conducted using different mouse genetic backgrounds and optimized ethanol exposure paradigms, the magnitude of injury should not be directly compared across models; conclusions are based on WT vs. KO comparisons within each model. These results also suggest that the relative sensitivity of the alcohol-mediated hippocampal damage in *Aldh2*-KO mice [46] compared to changes in cerebellum-associated locomotor tests could result from the hippocampi’s lower antioxidant capacity, often being implicated in aging and neurodegeneration [76,77], relative to that of the cerebellum. These results show greater abundance of several PTMs in pooled *Aldh2*-KO samples after ethanol exposure than their corresponding WT mice and are consistent with reduced aldehyde detoxification, as reported [66]; however, the findings do not establish the responsible metabolic pathway.

The two-way ANOVA results clarify whether ethanol effects were genotype dependent. Significant genotype × ethanol interactions in the *Cyp2e1*-KO FJC and rotarod datasets indicate that ethanol affected WT and *Cyp2e1*-KO mice differently, supporting a protective effect of Cyp2e1 deletion against ethanol-associated cerebellar neurodegeneration and motor impairment. In contrast, the *Aldh2*-KO FJC dataset showed significant main effects of genotype and ethanol treatment without a significant interaction, indicating that *Aldh2*-KO mice had greater overall FJC staining and ethanol increased overall neuronal damage, but the magnitude of the ethanol response was not statistically different between WT and *Aldh2*-KO mice. However, higher ethanol doses comparable to those used in the *Cyp2e1*-KO model were not examined in *Aldh2*-KO mice because Aldh2 deficiency increases susceptibility to ethanol toxicity, and such dosing would be expected to cause excessive morbidity or mortality rather than interpretable cerebellar injury outcomes.

At the cellular level, alcohol-mediated cerebellar damage is likely influenced by differential vulnerability among different neuronal cell populations, including Purkinje cells and granule neurons, which differ in metabolic demand and antioxidant capacity. Among the molecular regulators of cellular stress responses, sirtuins (SIRT1–7) are NAD^+^-dependent deacetylases involved in regulating cellular homeostasis [78,79]. In the liver, chronic alcohol consumption has been associated with a decreased NAD^+^/NADH ratio with the inhibition of SIRT1 and SIRT3, leading to impaired deacetylation of proteins, mitochondrial dysfunction, and steatosis [30,50].

However, in the present study, cerebellar analysis revealed no descriptive changes in the expression of sirtuin isoforms following binge alcohol exposure in either WT or *Cyp2e1*-KO mice. This lack of change in Sirt isoforms may reflect minimal alterations in NAD^+^ availability within the CNS, potentially due to the limited role of ADH-mediated ethanol metabolism in the brain [15,80]. These results suggest that the increased protein acetylation observed after binge alcohol exposure is not primarily associated with reduced total sirtuin protein expression in the cerebellum. This negative finding is still informative because it contrasts with alcohol-related changes reported in the liver [30,50,51] and highlights potential tissue-dependent differences in alcohol-mediated acetylation regulation. However, because only total sirtuin protein levels were assessed, future studies evaluating sirtuin activity, NAD^+^/NADH status, and other acetylation-regulating enzymes will be needed to define the mechanism more precisely.

In contrast, reactive aldehydes, such as acetaldehyde [81] and acrolein [82], are highly capable of forming covalent adducts with numerous proteins, leading to irreversible functional impairment and tissue injury in multiple organs, including the intestine, liver, and brain [81,83,84]. The accumulation of acrolein adducts observed in WT but not *Cyp2e1*-KO mice underscores the potential causal role of aldehyde-mediated PTMs in cerebellar injury. In addition, elevated accumulation of acrolein adducts in alcohol-exposed *Aldh2*-KO mice compared to WT mice is likely to contribute to cerebellar damage, particularly in neuronal populations with high metabolic demand.

Given the central role of CYP2E1 in promoting oxidative stress and PTM accumulation, targeting this enzyme represents a promising therapeutic strategy for mitigating alcohol-induced cerebellar degeneration. Pharmacological inhibition of CYP2E1 has been explored using compounds that either coordinate with the heme iron of the enzyme or act as competitive or noncompetitive inhibitors with respect to ethanol and other substrates [85]. Naturally derived compounds such as diallyl sulfide (from garlic) [86], piperine (from black pepper) [87], berberine (a plant-derived alkaloid) [88], indole-3-carbinol (from cabbage or apple) [89], and ellagic acid (from pomegranate) [90] have demonstrated CYP2E1-inhibitory and antioxidant properties, with reported protective and anticancer effects in many tissues. However, the efficacy and safety of these exogenous compounds in the context of alcohol-induced neurotoxicity require further investigation.

In parallel, the defensive role of mitochondrial ALDH2 warrants significant attention. ALDH2 metabolizes acetaldehyde and other toxic carbonyl species into less harmful metabolites, thereby limiting aldehyde-induced oxidative stress and PTMs. Enhancing ALDH2 activity could mitigate acetaldehyde- and acrolein-induced neurotoxicity, whereas loss or inhibition of ALDH2, as demonstrated in *Aldh2*-KO mice, exacerbates alcohol-induced cerebellar damage. Studies have shown that pharmacological activation of ALDH2 using Alda-1 and related structural derivative compounds reduces oxidative stress and neuronal injury in experimental models, including alcohol-associated brain injury [43,91]. As shown in this study, CYP2E1 and ALDH2 exert opposing influences on cerebellar vulnerability during binge alcohol exposure, with CYP2E1 amplifying oxidative injury and ALDH2 functioning as a critical defensive buffer. These genetic findings provide a rationale for future studies testing a dual-target approach based on CYP2E1 inhibition and ALDH2 activation. However, because no CYP2E1 inhibitor or ALDH2 activator was evaluated in the present study, the current results do not establish pharmacological efficacy or therapeutic benefit. CYP2E1 inhibition and ALDH2 activation should therefore be considered candidate strategies for future preclinical investigation.

The complementary genetic, histological, biochemical, and behavioral findings provide consistent evidence for the opposing roles of CYP2E1 and ALDH2 in cerebellar responses to binge alcohol; however, several limitations define the scope of their interpretation. Cerebellar ethanol, acetaldehyde, propionaldehyde, 4-HNE, and related metabolites, together with CYP2E1, ALDH2, and other ethanol-metabolizing enzyme activities, were not directly quantified. Direct metabolic studies will therefore be valuable for distinguishing the relative contributions of acetaldehyde, CYP2E1-derived ROS, catalase, ADH, and other CYP/ALDH pathways. The measured PTMs provide biologically relevant evidence of oxidative and aldehyde-associated stress but represent indirect metabolic readouts. Similarly, FJC staining sensitively identifies degenerating cells but does not specify the mechanism of cell death or distinguish Purkinje cells, granule cells, and other cerebellar cell populations, while rotarod performance complements the tissue findings as an integrated motor assessment rather than a cerebellum-specific test. Pooled lysates allowed group-level biochemical characterization but limited assessment of animal-to-animal variability. In addition, based on our prior observations [46,64], the *Cyp2e1*- and *Aldh2*-KO experiments were independently optimized using different genetic backgrounds, ethanol doses, animal ages and sources, and assessment intervals. Conclusions are therefore based on WT-vs-KO comparisons within each model rather than direct comparisons of effect magnitude between the two mouse strain models. The use of separate endpoint cohorts, female mice only, non-littermate Aldh2 groups, and genetic rather than pharmacological manipulation should also be considered. Future studies incorporating direct metabolite and enzyme-activity measurements, individual biochemical samples, cell-specific analyses, both sexes, matched experimental designs, and pharmacological CYP2E1 inhibition and/or ALDH2 activation will further extend the present findings. 

Despite these considerations, to our knowledge, this is the first study to investigate within a single experimental framework for the opposing roles of CYP2E1 and ALDH2 in the context of binge alcohol-mediated cerebellar injury using their respective knockout mouse models. Cyp2e1 deletion was associated with reduced ethanol-induced FJC-positive degeneration, lower accumulation of selected PTMs, preservation of VDAC abundance, and protection against ethanol-related rotarod impairment. In contrast, Aldh2 deletion was associated with greater overall FJC positivity and increased accumulation of oxidative and aldehyde-associated PTMs. Together, these complementary findings support an injury-promoting role for CYP2E1 and a protective role for ALDH2 in the cerebellum. The term “opposing roles” describes the direction of their biological influences within the respective models but does not necessarily imply equivalent effect magnitude or a direct quantitative comparison between the independently conducted experiments. By extending the understanding of alcohol-metabolizing pathways beyond the liver to the cerebellum, this study identifies CYP2E1 and ALDH2 as important determinants of cerebellar vulnerability to binge alcohol exposure and provides a foundation for future mechanistic and therapeutic investigations targeting these pathways.

## 4. Materials and Methods

### 4.1. Animal Treatment

All experimental animal procedures were approved by the National Institute on Alcohol Abuse and Alcoholism (NIAAA) Institutional Animal Care and Use Committee (Protocol Number: LMBB-BS-1, approved on 27 February 2023). Young (2–4 months old) female WT and global *Cyp2e1*-KO mice on the Svj/129 background strain (both originally obtained from Dr. Frank J. Gonzales, National Cancer Institute and further bred internally) [64] were maintained separately by homozygous breeding of each mouse strain under tightly controlled lighting (12 h light/dark cycle), temperature (22  ±  3 °C), and humidity (45–55%), with free access to rodent chow and water. Age- and sex-matched mice (*n* = 4~6/group) were randomly selected and administered via oral gavage with ethanol at 5 g/kg/dose (30% ethanol *w*/*v* with a gavage volume of 0.51 mL/25 g mice) for up to 7 consecutive doses at 12 h intervals. Cerebellar tissues were collected 1 h after the last dose of ethanol treatment for biochemical and confocal analyses. Control mice were given dextrose (0.5 g/mL at a gavage volume of 0.44 mL/25 g mice) to achieve an energy effect similar to that of EtOH administration. Another group of mice (5–7 months old) were exposed to 5 consecutive ethanol doses (5 g/kg/dose at 12 h intervals) and then subjected to the rotarod locomotor activity test 48 h after the last ethanol dose.

In comparison, to study the protective role of ALDH2, young (2–4 months old) female WT and global *Aldh2*-KO mice (*n* = 4~6/group) on C57BL/6J background (originally obtained from Dr. Toshihiro Kawamoto, University of Occupational and Environmental Health and further internally bred) [66] and age-, sex-, and strain-matched C57BL/6J WT mice (purchased from Jackson Laboratory, Bar Harbor, ME, USA) were randomly selected and exposed to 3 consecutive doses of ethanol at 4 g/kg/dose via oral gavage at 12 h intervals. In this experiment, cerebellar tissues were collected at 1 h after the last dose of ethanol and analyzed by immunoblots and confocal microscopy with Fluoro-Jade C (FJC) staining for neurodegeneration, as reported [46]. Again, control mice were given dextrose instead to achieve an energy effect similar to that of EtOH administration. To assess the role of ALDH2 in locomotor function, another set of WT and *Aldh2*-KO young (2–4-month-old) mice was subjected to the rotarod test 72 h after the last of the five consecutive doses of ethanol (4 g/kg/dose) at 12 h intervals.

Ethanol exposure paradigms were selected based on the previously reported differential sensitivity of these KO models to alcohol-mediated tissue injury. *Aldh2*-KO mice are more susceptible to alcohol-mediated tissue injury than corresponding WT mice [46,66]; therefore, a lower binge ethanol dose of 4 g/kg/dose was used to evaluate the increased susceptibility of *Aldh2*-KO mice to alcohol-mediated cerebellar damage. In contrast, the higher binge ethanol dose of 5 g/kg/dose used in the *Cyp2e1*-KO experiment [64] was selected to establish a robust cerebellar injury phenotype in WT mice and determine whether Cyp2e1 deletion confers resistance to alcohol-mediated cerebellar damage compared with corresponding WT mice.

The 1 h post-ethanol time point for tissue collection was selected to capture early alcohol-induced molecular and histological changes in the cerebellum. In contrast, rotarod testing was performed 48–72 h after the last ethanol dose to evaluate delayed cerebellum-associated motor coordination and balance deficits after the acute intoxication period.

### 4.2. Fluoro-Jade-C Staining

The frozen cerebellum tissues, of individual animals, from WT or *Cyp2e1*-KO mice before and after binge alcohol exposures, as indicated, were subjected to FJC staining, as reported previously [46]. The FJC-mediated fluorescence images were analyzed by using a confocal microscope (Carl Zeiss LSM 700, Oberkochen, Germany). Similar confocal analyses of FJC staining were conducted with the respective set of the WT and *Aldh2*-KO mice before and after binge alcohol exposure [46]. Quantification of FJC images was done with ImageJ 1.54k (NIH). The images’ channels were split, focusing on the green channel, the background was subtracted at a value of 30, and then a threshold behind the first peak was found to target the brightest neurons. This threshold value was then used consistently across each comparison. Lastly, image processing through selecting “binary function,” then “open”, to remove noise, and imaging artifacts were removed. FJC-positive damaged neurons were then counted via the “analyzing particles” function.

### 4.3. Immunoblotting

Cerebellar extracts from mice within the same groups (*n* = 4~6/group) were pooled and homogenized with 1x RIPA buffer. Bradford protein assays were conducted to quantify protein concentrations, and then equal amounts of protein were used for SDS/PAGE followed by transfer to nitrocellulose membranes. Specific primary and secondary antibodies were then used to detect target PTMs and proteins, as listed in Table 1. Imaging target proteins was carried out using enhanced chemiluminescence (ECL) substrates (Thermo-Fisher Scientific, Waltham, MA, USA, #34095) and an iBright FL1500 imager (Thermo-Fisher Scientific, Carlsbad, CA, USA). The resulting immunoreactive band intensities were quantified by densitometry with the ImageJ application (NIH), as previously described [46].

### 4.4. Preparation of Mitochondrial Fraction

Frozen cerebellum samples were homogenized with cold 250 mM sucrose, 50 mM Tris, pH 7.4, and 1 mM EGTA (STE) buffer [26]. Cerebellar extracts were then spun at 500× *g* to remove cell debris and nuclear fraction, followed by spinning at 13,000× *g* at 4 °C for 10 min to prepare mitochondrial pellets. The solubilized fractions, enriched after three cycles of quick freeze–thaw of the washed mitochondrial pellets in a hypotonic buffer, were used for immunoblot analysis.

### 4.5. Rotarod Locomotor Assessment

Up to five mice were placed on the rotarod apparatus, Med Associates Inc. (St. Albans, VT, USA). ENV-574M five-lane rotarod for mice, simultaneously for each trial. Mice were given one training session with a speed setting of 3.5–35 RPM. During the pre-test training, all mice were allowed to remount the rotarod an unlimited number of times until the 300 s threshold was reached. Following initial pre-test training, performance testing was conducted, and the duration each mouse remained on the rotating rod before falling or reaching 350 s was recorded. Performance was reported as latency to fall in seconds by following the method, as reported elsewhere [45].

### 4.6. Statistical Analysis

Statistical analyses were performed using GraphPad Prism version 10.3.1 (GraphPad Software, San Diego, CA, USA). Data obtained from individual animals, including Fluoro-Jade C-positive cell counts (from ImageJ) and rotarod latency-to-fall measurements, were analyzed by ordinary two-way ANOVA with genotype and ethanol treatment/exposure as fixed factors. When post hoc pairwise comparisons were performed, Tukey’s multiple-comparisons test was used. Main effects of genotype, ethanol treatment/exposure, and genotype × ethanol treatment/exposure interactions are reported in Supplementary Table S1A. Tukey-adjusted post hoc pairwise comparisons can be found in Supplementary Table S1B. Data are presented as the mean ± SD, and *p* < 0.05 was considered statistically significant (*p* < 0.01 (**), *p* < 0.001 (***), and *p* < 0.0001 (****)).

For immunoblot experiments in which cerebellar lysates were pooled within each experimental group, densitometry was used to describe representative group-level biochemical changes and technical reproducibility; these pooled immunoblot data were not treated as independent biological replicates for inferential statistical testing.

## Figures and Tables

**Table 1:** Antibodies used with dilution and company information.

Antibody	Dilution (-Fold)	Company
Acetyl-Lysine	1:4000×	Cell Signaling
Phosphorylated Ser/Thr-Pro	1:5000×	Abcam
Acrolein adducts	1:1000×	Abcam
VDAC	1:1000×	Abcam
GAPDH	1:8000×	Cell Signaling
3-NT	1:2000×	Abcam
ALDH2	1:3000×	Abcam
Sirtuin-1	1:1000×	Cell Signaling
Sirtuin-2	1:1000×	Cell Signaling
Sirtuin-3	1:1000×	Cell Signaling
Sirtuin-4	1:1000×	Cell Signaling
Sirtuin-5	1:1000×	Cell Signaling
Sirtuin-6	1:1000×	Cell Signaling
Sirtuin-7	1:1000×	Cell Signaling

## Data Availability

All raw data are kept in the lab as per NIH guidelines and will be available upon request.

## Supplementary Materials

The following supporting information can be downloaded at: https://www.mdpi.com/article/10.3390/ijms27156825/s1.

## Abbreviations

The following abbreviations are used in this manuscript:

CYP2E1	Cytochrome P450-2E1
ALDH2	Mitochondrial aldehyde dehydrogenase 2
WT	Wild-type
KO	Knockout
EtOH	Ethanol
FJC	Fluoro-jade C
PTMs	Post-translational modifications
ADH	Alcohol dehydrogenase
AUD	Alcohol use disorder
ROS	Reactive oxygen species
iNOS	Inducible nitric oxide synthase
NO	Nitric oxide
DAPI	4′,6′-diamidino-2-phenyl indole
NAD^+^	Nicotinamide adenine dinucleotide
SIRTs	Sirtuin isoforms
MDA	Malondialdehyde
ACR	Acrolein
4-HNE	4-hydroxynonenal
ALD	Alcohol-associated liver disease
ARBD	Alcohol-related brain damage
ECL	Enhanced chemiluminescence
STE	Sucrose-Tris-EGTA buffer
3-NT	3-Nitrotyrosine (nitrated)
Ac-Lys	Acetylation
p-S/T-Pro	Phosphorylated serine/threonine residues
mt	Mitochondrial fractions
GAPDH	Glyceraldehyde 3-phosphate dehydrogenase
VDAC	Voltage-dependent anion channel
CNS	Central nervous system
