# Supplementary material for: Opposing Roles of CYP2E1 and ALDH2 in Binge Alcohol-Mediated Cerebellar Damage
Source: Int J Mol Sci. 2026 Jul 30;27(15):6825. doi: 10.3390/ijms27156825 (PMC13466411; doi:10.3390/ijms27156825)
Supplement: Supplementary file 1 [file ijms-27-06825-s001.zip › ijms-4445963-supplementary.pdf]

Supplementary Table S1A. Two-way ANOVA and Tukey's multiple-comparisons analysis of individual-animal datasets

Figure / Dataset	Endpoint	n	Genotype main effect	Ethanol treatment / dose main effect	Genotype × treatment interaction
Fig. 1, Cyp2e1-KO FJC staining	FJC-positive damaged neuron count	n = 3/group	F(1, 12) = 83.61, p < 0.0001	F(2, 12) = 37.30, p < 0.0001	F(2, 12) = 38.92, p < 0.0001
Fig. 3, Cyp2e1-KO rotarod	Latency to fall, seconds	Control WT: n = 6; Alcohol WT: n = 5; Control Cyp2e1-KO: n = 6; EtOH Cyp2e1-KO: n = 7	F(1, 20) = 8.852, p = 0.0075	F(1, 20) = 17.26, p = 0.0005	F(1, 20) = 16.00, p = 0.0007
Fig. 4, Aldh2-KO FJC staining	FJC-positive damaged neuron count	n = 3/group	F(1, 8) = 16.64, p = 0.0035	F(1, 8) = 5.464, p = 0.0476	F(1, 8) = 1.111, p = 0.3226

Legend: n indicates the number of individual animals analyzed per group; FJC, Fluoro-Jade C; EtOH, ethanol; KO, knockout; WT, wild-type

Supplementary Table S1B. Tukey-adjusted post hoc pairwise comparisons

Figure / Dataset	Tukey pairwise comparison	Mean difference	95% CI of difference	Adjusted p-value	Significance
**Fig. 1, Cyp2e1-KO FJC**	Control:WT vs Control:Cyp2e1-KO	4.667	-14.28 to 23.61	0.9565	ns
**Fig. 1, Cyp2e1-KO FJC**	Control:WT vs EtOH-3:WT	-4.667	-23.61 to 14.28	0.9565	ns
**Fig. 1, Cyp2e1-KO FJC**	Control:WT vs EtOH-7:WT	-62.33	-81.28 to -43.39	<0.0001	****
**Fig. 1, Cyp2e1-KO FJC**	Control:Cyp2e1-KO vs EtOH-3:Cyp2e1-KO	5.333	-13.61 to 24.28	0.9265	ns
**Fig. 1, Cyp2e1-KO FJC**	Control:Cyp2e1-KO vs EtOH-7:Cyp2e1-KO	3.000	-15.95 to 21.95	0.9936	ns
**Fig. 1, Cyp2e1-KO FJC**	EtOH-3:WT vs EtOH-3:Cyp2e1-KO	14.67	-4.279 to 33.61	0.1706	ns
**Fig. 1, Cyp2e1-KO FJC**	EtOH-3:WT vs EtOH-7:WT	-57.67	-76.61 to -38.72	<0.0001	****
**Fig. 1, Cyp2e1-KO FJC**	EtOH-3:Cyp2e1-KO vs EtOH-7:Cyp2e1-KO	-2.333	-21.28 to 16.61	0.9980	ns
**Fig. 1, Cyp2e1-KO FJC**	EtOH-7:WT vs EtOH-7:Cyp2e1-KO	70.00	51.05 to 88.95	<0.0001	****
**Fig. 3, Cyp2e1-KO rotarod**	Control:WT vs Control:Cyp2e1-KO	39.33	-111.5 to 190.1	0.8839	ns

**Fig. 3, Cyp2e1-KO rotarod**	Control:WT vs Alcohol:WT	312.9	154.7 to 471.0	0.0001	***
**Fig. 3, Cyp2e1-KO rotarod**	Control:Cyp2e1-KO vs Alcohol:Cyp2e1-KO	5.905	-139.4 to 151.2	0.9995	ns
**Fig. 3, Cyp2e1-KO rotarod**	Alcohol:WT vs Alcohol:Cyp2e1-KO	-267.6	-420.6 to -114.7	0.0005	***
**Fig. 4, Aldh2-KO FJC**	Control:WT vs Control:Aldh2-KO	-11.00	-27.47 to 5.468	0.2200	ns
**Fig. 4, Aldh2-KO FJC**	Control:WT vs EtOH-3:WT	-4.667	-21.13 to 11.80	0.8017	ns
**Fig. 4, Aldh2-KO FJC**	Control:WT vs EtOH-3:Aldh2-KO	-23.33	-39.80 to -6.866	0.0082	**
**Fig. 4, Aldh2-KO FJC**	Control:Aldh2-KO vs EtOH-3:Aldh2-KO	-12.33	-28.80 to 4.134	0.1547	ns
**Fig. 4, Aldh2-KO FJC**	EtOH-3:WT vs EtOH-3:Aldh2-KO	-18.67	-35.13 to -2.199	0.0275	*

Legend: FJC, Fluoro-Jade C; EtOH, ethanol; KO, knockout; WT, wild-type; not significant (ns), p<0.05 (*), p<0.01 (**), p<0.001 (***), and p<0.0001 (****)

Supplementary Figures to show the full immunoblot images

Figure 2	Immunoblots
Acetyl-Lys	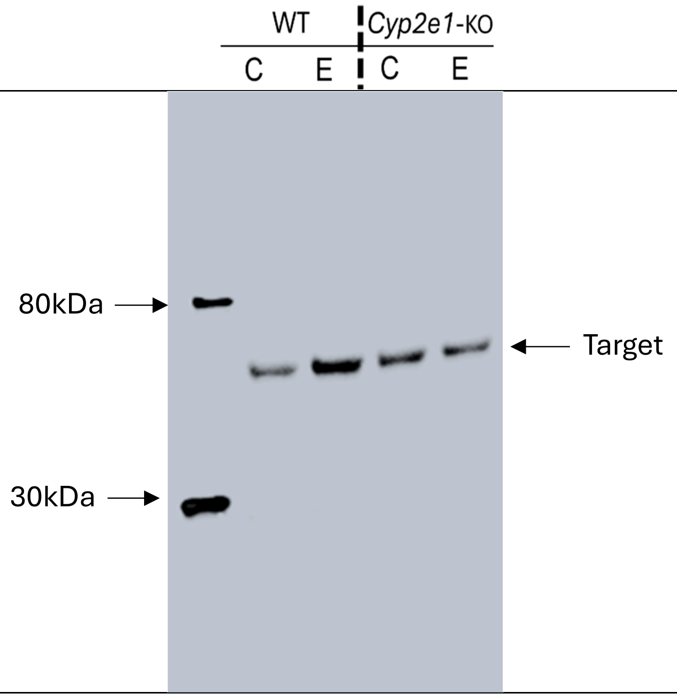

p-S/T-Pro

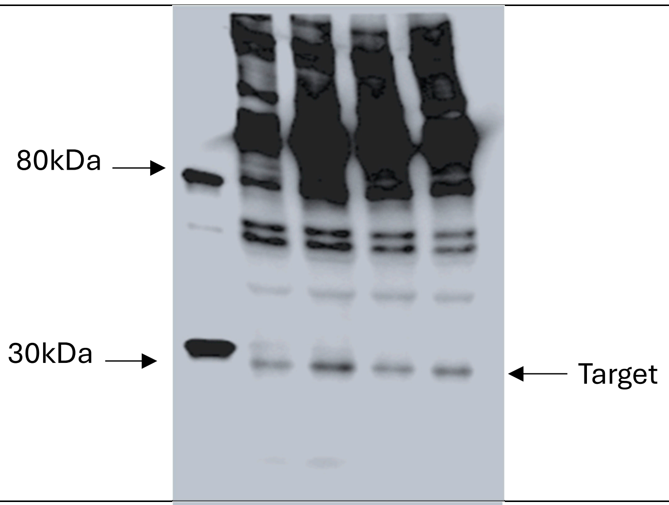

mt. p-S/T-Pro

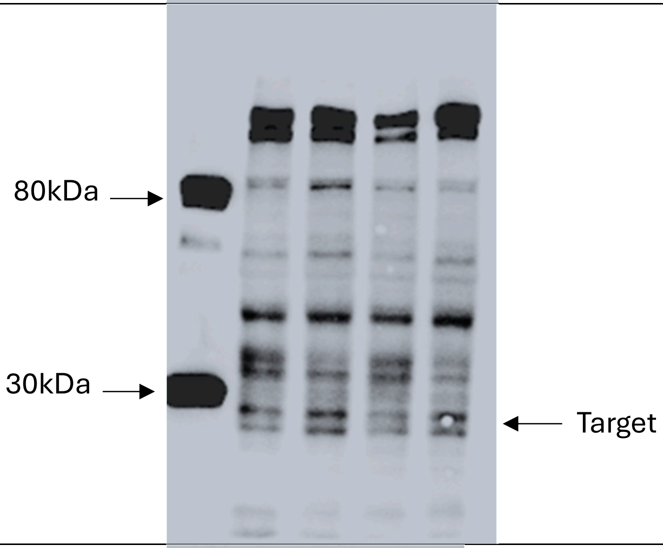

Acrolein Adducts

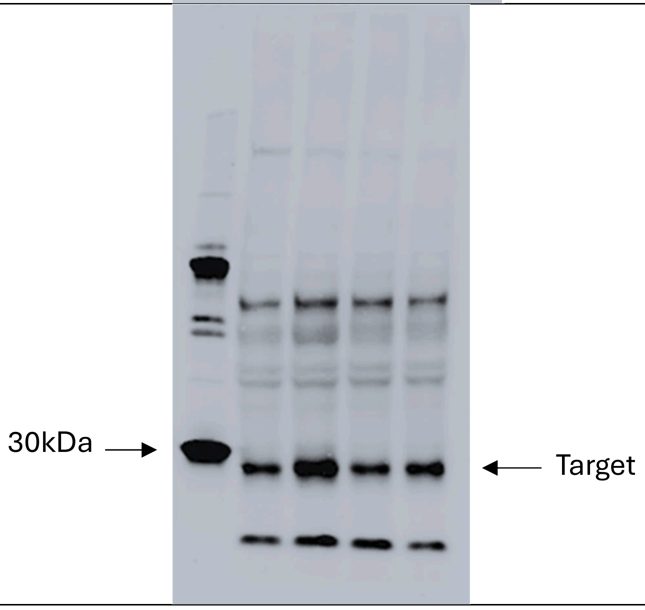

<table>
<tr><td>mt. Acrolein Adduct</td><td>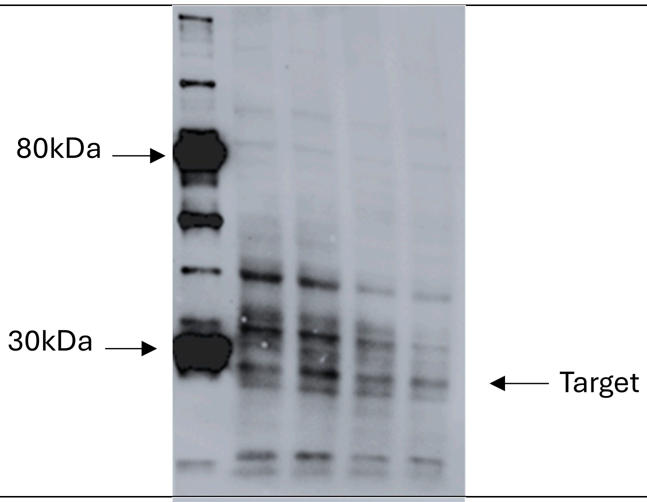
</td></tr>
<tr><td>mt. VDAC</td><td>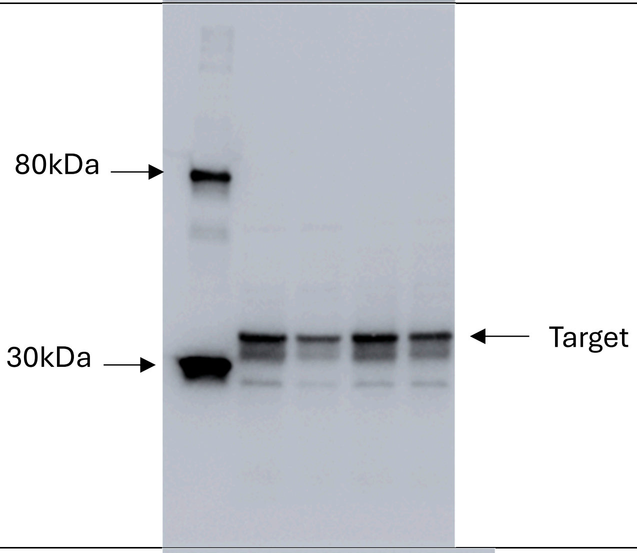
</td></tr>
<tr><td>GAPDH</td><td>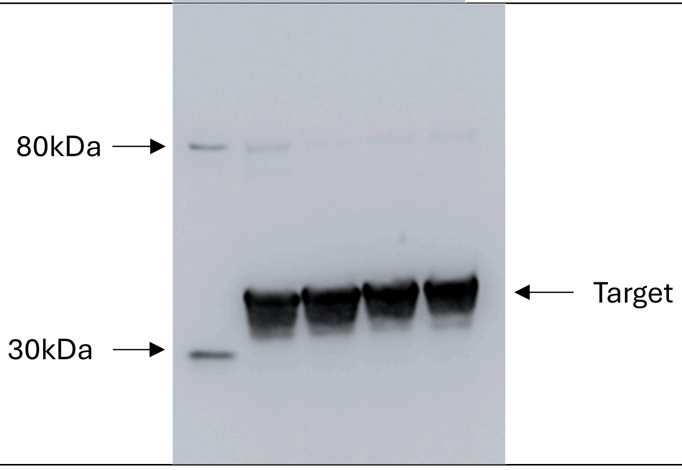
</td></tr>
</table>

mt. GAPDH	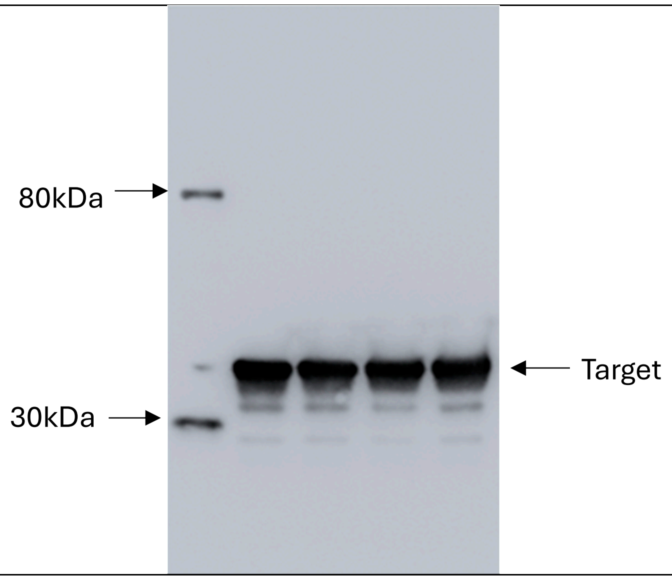

Figure 5	Immunoblots
	WT / *Aldh2*-KO<br>C E / C E
3-NT	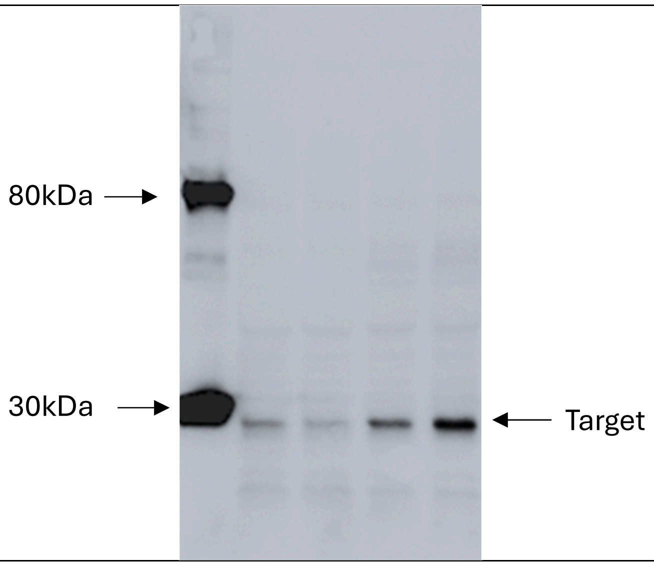

<table>
<tr><td>p-S/T-Pro</td><td>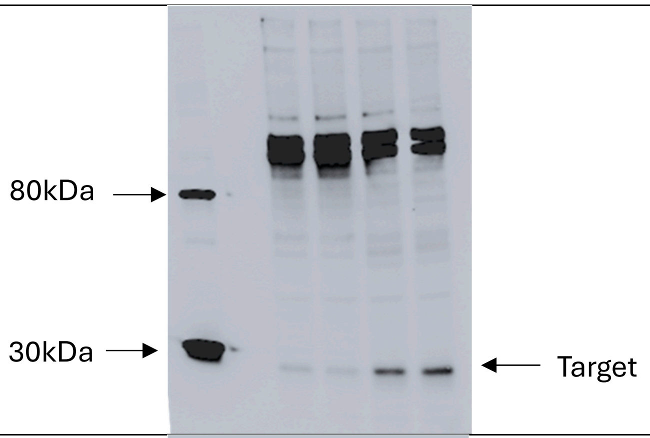
</td></tr>
<tr><td>Acrolein Adduct</td><td>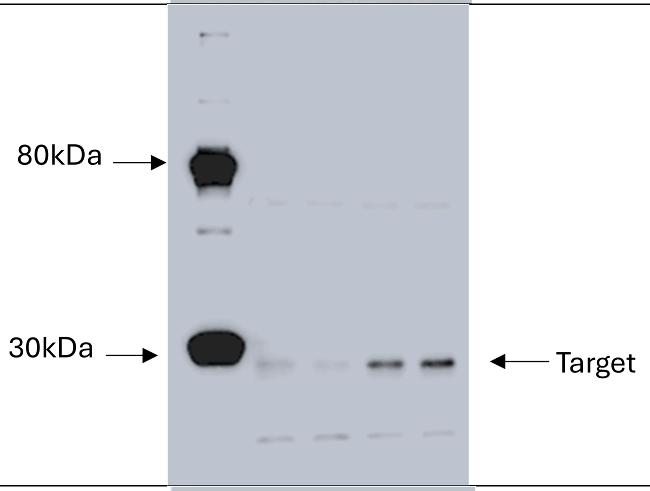
</td></tr>
<tr><td>Acetyl-Lysine</td><td>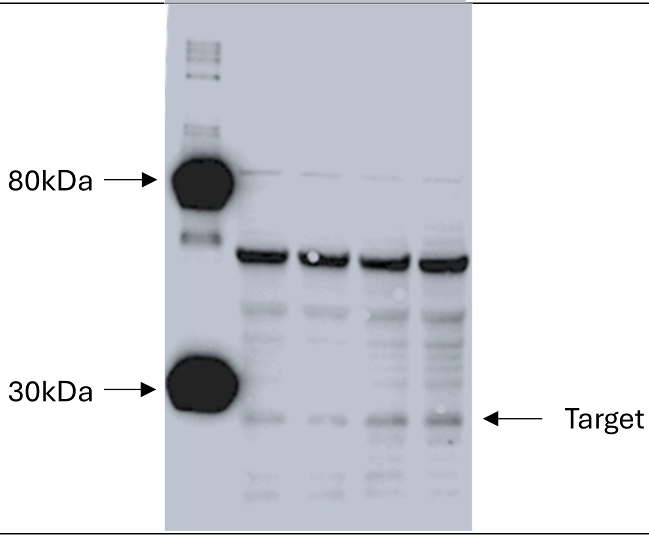
</td></tr>
</table>

<table>
<tr><td>GAPDH</td><td>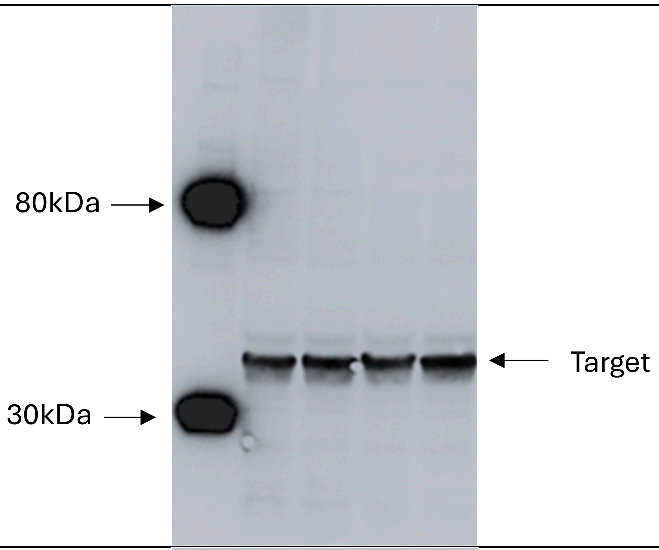
</td></tr>
<tr><td>ALDH2</td><td>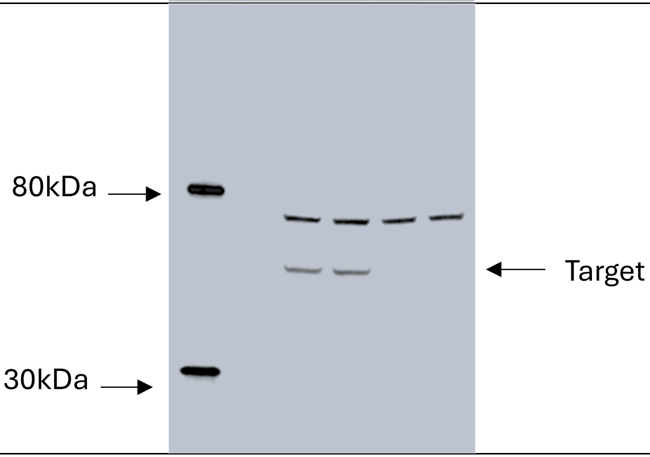
</td></tr>
</table>

Figure 6	Immunoblots
	WT | *Cyp2e1*-KO<br>C E | C E

<table>
<tr><td>SIRT1</td><td>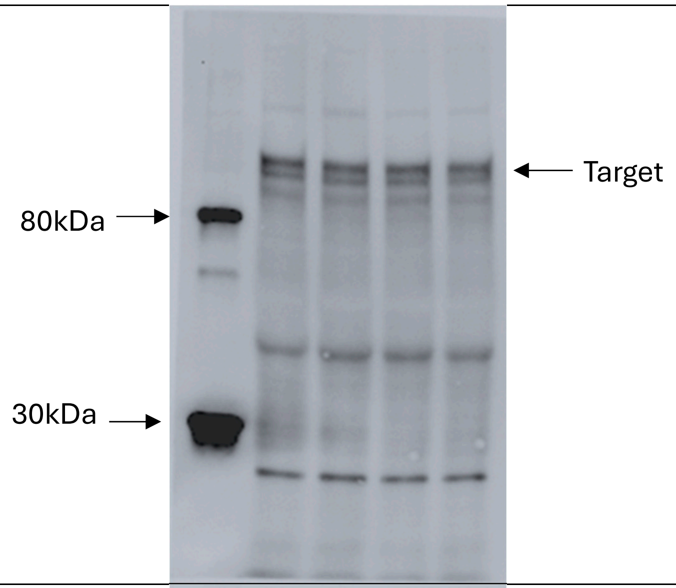
</td></tr>
<tr><td>SIRT2</td><td>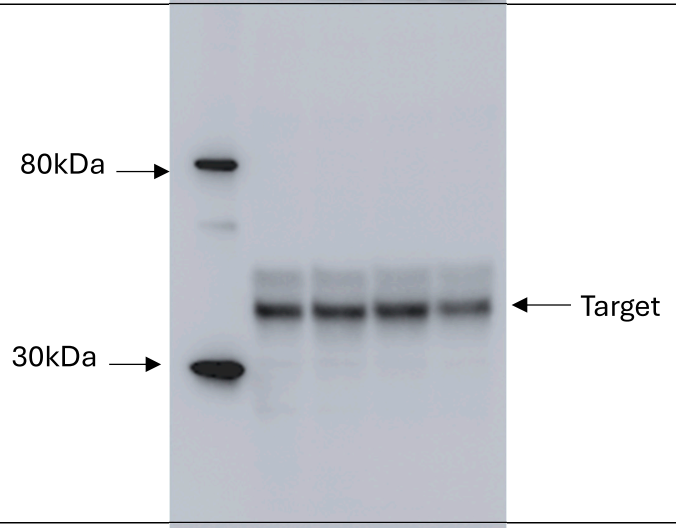
</td></tr>
<tr><td>SIRT3</td><td>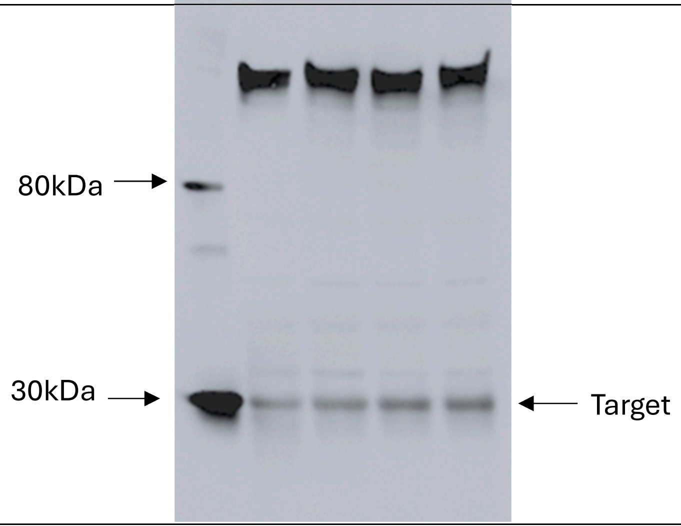
</td></tr>
</table>

SIRT4	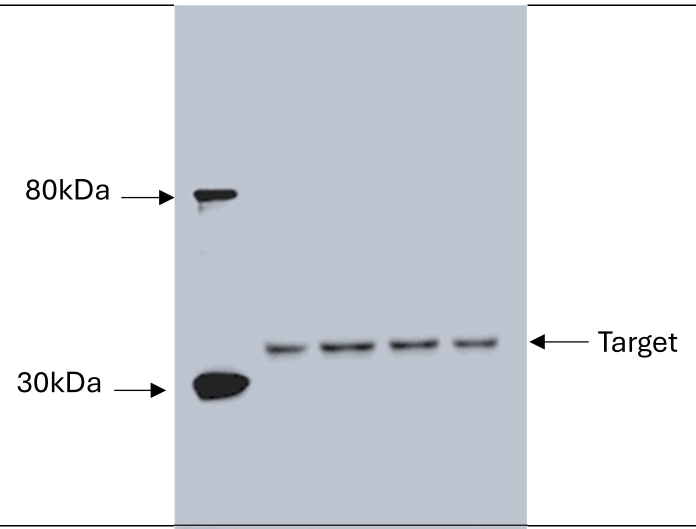
SIRT5	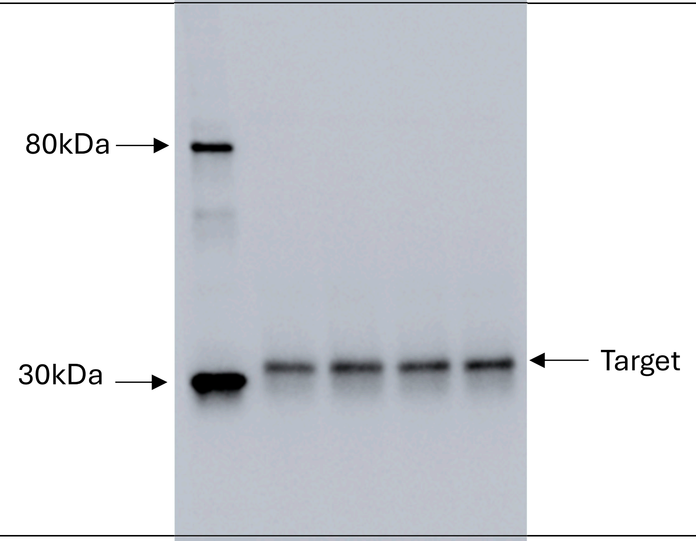
SIRT6	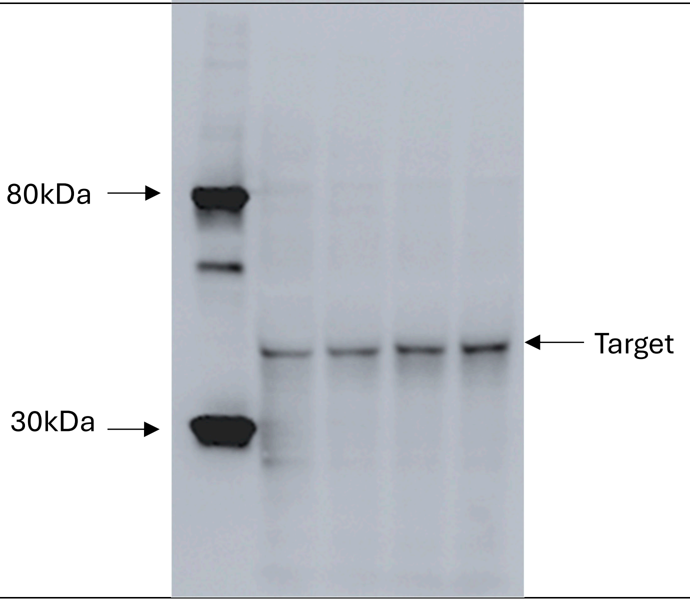

SIRT7	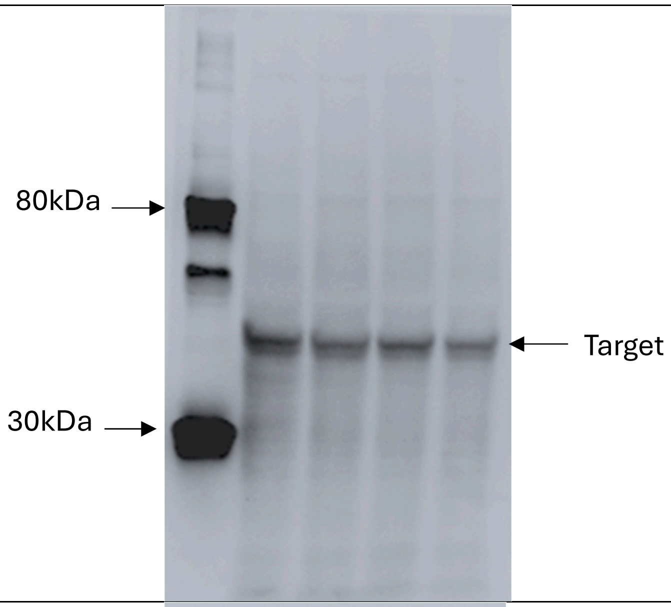
GAPDH	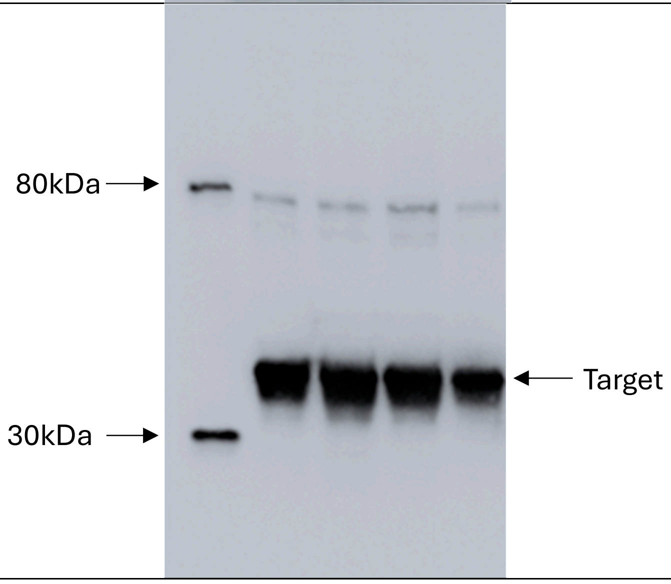
